# Glutamate Receptors in Alzheimer's Disease: Mechanisms and Therapies

**DOI:** 10.1155/2016/8256196

**Published:** 2016-05-16

**Authors:** Victor Anggono, Li-Huei Tsai, Jürgen Götz

**Affiliations:** ^1^Clem Jones Centre for Ageing Dementia Research, Queensland Brain Institute, The University of Queensland, Brisbane, QLD 4072, Australia; ^2^Picower Institute for Learning and Memory, Massachusetts Institute of Technology (MIT), Cambridge, MA 02139, USA; ^3^Department of Brain and Cognitive Sciences, MIT, Cambridge, MA 02139, USA; ^4^Broad Institute of MIT and Harvard, Cambridge, MA 02139, USA

Alzheimer's disease (AD) manifests as a progressive loss in memory, cognition, and language that is commonly associated with elevated levels of amyloid-beta (A*β*) peptide and hyperphosphorylated tau in the brain. There is currently no cure for AD as its causes remain poorly understood. Accumulating evidence suggests that synaptic dysfunction is a major contributor early in disease pathogenesis prior to neuronal loss. Glutamatergic neurotransmission is particularly vulnerable to the neurotoxic effects of various assemblies of A*β* and hyperphosphorylated tau. Indeed, these toxic species act in synergy and severely disrupt excitatory synaptic transmission, synaptic plasticity, and network activity.

The chronology of how A*β* impairs neuronal function is not completely understood. However, accumulating evidence suggests that A*β* causes an initial hyperexcitability of neurons due to spillover and excessive levels of glutamate in the extrasynaptic space, leading to overstimulation of NMDA receptors and subsequently synaptic loss and cell death. The clearance of extracellular glutamate is primarily accomplished by astrocytic glutamate transporters, a process that is disrupted by A*β*. In this issue, Y.-L. Lan et al. present a new view that enhancing the interaction and synergy between aquaporin-4 and glutamate transporter-1 may confer a neuroprotective effect against excitotoxicity and neuronal death in AD.

Synaptic depression is a consequence of reduced numbers of glutamate receptors in the postsynaptic density and the eventual loss of synapses. S. Guntupalli et al. provide a comprehensive view of key signal transduction pathways underlying A*β*-induced endocytosis of the AMPA-type glutamate receptors, most of which are commonly shared with mechanisms that lead to long-term depression. Although the effects of A*β* in Hebbian-type synaptic plasticity, such as long-term potentiation and depression, are well established, its role in influencing homeostatic synaptic plasticity has only begun to emerge. S.-S. Jang and H.-J. Chung discuss the idea that disruption of Hebbian plasticity in AD is due to aberrant metaplasticity, a form of homeostatic plasticity that sets the threshold for future synaptic plasticity. In addition, they provide comprehensive views on the mechanisms underlying aberrant metaplasticity in AD.

Actin cytoskeleton and synaptic adhesion molecules play crucial roles in maintaining the integrity and structural plasticity of synapses. H. Stefen et al. focus on the actin cytoskeleton and discuss the current understanding of how A*β* disrupts actin dynamics, leading to impairments in the trafficking of AMPA, NMDA, and metabotropic glutamate receptors in the postsynaptic compartment. Several classes of synaptic adhesion molecules are known to mediate the attachment of presynaptic and postsynaptic membranes to ensure that the synaptic junction stays intact during synaptic plasticity. I. Leshchyns'ka and V. Sytnyk offer scientific evidence to illustrate that A*β* interacts with synaptic adhesion molecules and affects their expression and synaptic localization, all of which impair the function and integrity of synapses, leading to the disruption of neuronal networks in AD.

The striatal-enriched tyrosine phosphatase (STEP) plays a key role in regulating AMPA and NMDA receptor trafficking in synaptic plasticity. M. Kamceva et al. provide an update on recent advances in this area, including a discussion of the substrates and signaling pathways of STEP associated with A*β* toxicity, as well as the potential therapeutic benefits of STEP inhibition in mouse models of AD. S.-S. Jang et al. have also investigated the effects of epileptic seizures, which are commonly associated with AD, in regulating the expression of STEP and its substrates. They found that a single episode of electroconvulsive seizures potently increases STEP expression, concomitant with decreases in the levels of NMDA receptors and dephosphorylation of extracellular signal regulated kinase 1/2 (ERK1/2). Interestingly, electroconvulsive seizures also increase A*β* production. This study suggests that seizure-induced upregulation of A*β* and STEP leads to NMDA receptor internalization and ERK1/2 inactivation in the hippocampus.

By highlighting the molecular mechanisms that underlie A*β*-induced defects in glutamate receptor trafficking, synaptic plasticity, and network activity, we hope that this special issue will provide a better mechanistic understanding of the etiology of the cognitive and memory deficits associated with AD. We also trust that this special issue will spark further research that will contribute to ongoing efforts to identify meaningful therapeutic targets for the treatment of AD.

*Victor Anggono* *Victor Anggono* *Li-Huei Tsai* *Li-Huei Tsai* *Jürgen Götz* *Jürgen Götz*

